# Executive Function Performance in Young Adults When Cycling at an Active Workstation: An fNIRS Study

**DOI:** 10.3390/ijerph16071119

**Published:** 2019-03-28

**Authors:** Tao Huang, Qian Gu, Zhangyan Deng, Chilun Tsai, Yue Xue, Jimeng Zhang, Liye Zou, Zuosong Chen, Kun Wang

**Affiliations:** 1 Department of Physical Education, Shanghai Jiao Tong University, Shanghai 200240, China; taohuang@sjtu.edu.cn (T.H.); sjtuguqian@sjtu.edu.cn (Q.G.); zhangyandeng@sjtu.edu.cn (Z.D.); henry41202@yahoo.com.tw (C.T.); xy0927@sjtu.edu.cn (Y.X.); simon2017@sjtu.edu.cn (J.Z.); zschen1971@126.com (Z.C.); 2 Graduate school of Education, Shanghai Jiao Tong University, Shanghai 200240, China; 3 Lifestyle (Mind-Body Movement) Research Center, College of Sports Science, Shenzhen University, Shenzhen 518060, China; liyezou123@gmail.com

**Keywords:** sedentary behavior, executive functions, active workstation, exercise, fNIRS

## Abstract

Background: This study aimed to investigate the effects of self-paced cycling at an active workstation on executive functions and cortical activity. Methods: In a crossover study design, 37 young adults (45.9% females) were randomly assigned to the following two task conditions: (1) performing cognitive tests during sitting, (2) performing cognitive tests while cycling at an active workstation. Executive functions were assessed by the Stroop color and word test and the task-switching paradigm. Cortical activity was monitored using a multi-channel functional near-infrared spectroscopy (fNIRS) system. Results: The behavioral results showed that there were no significant differences on the Stroop interference effects (*P* = 0.66) between the sitting and the cycling conditions. In all probability, no differences on the global switch costs (*P* = 0.90) and local switch costs (*P* = 0.67) were observed between the sitting and the cycling conditions. For the fNIRS results, the oxygenated hemoglobin (oxy-Hb) in response to the Stroop interference in channels 5, 10, and 12 were decreased during the cycling condition (all Ps < 0.05, FDR-corrected). Conversely, the oxy-Hb associated with the global switch costs in channels 3, 29, and 31 were increased during the cycling condition (all Ps < 0.05, FDR-corrected). Conclusions: The findings indicated that behavioral performances on executive functions were not affected by cycling at an active workstation, while cognitive resources were reallocated during cycling at an active workstation.

## 1. Introduction

Sedentary behavior refers to any activities when people are awake, where their energy expenditure is less than 1.5 MET (metabolic equivalent) [1]. It is widely accepted that sedentary behaviors and physical inactivity have adverse impacts on an individual’s health, but also bring about substantial medical and economic burden [2,3]. Such results have drawn increasing public attention in recent years. Prolonged sedentary time is independently associated with increased risk of developing noncommunicable diseases, such as obesity [4], diabetes [5], cardiovascular diseases [6], and mental disorders [7,8]. Conversely, breaking up sedentary time has been shown to contribute immensely to physical and mental health, such as favorable metabolic changes [9,10]. 

Young adults usually spend a great amount of sitting time at work or study in addition to other forms of sedentary behaviors. Therefore, it is especially urgent to intervene sedentary behaviors that are essential for chronic disease prevention in this age group. The recent evidence suggests that implementation of active workstations may be a promising intervention strategy for breaking up sedentary time at the workplace [11,12,13,14]. For example, active workstations provided the opportunity to perform low-intensity exercise or to stand while working or studying, which led to reduced sedentary time [15,16]. Furthermore, it consistently demonstrated that active workstations improved anthropometric indicators (e.g., body weight [17,18] and waist circumstances [19]), cardiovascular metabolic risk indicators (e.g., cholesterol [19], high-density lipoprotein [20], and postprandial blood glucose [21]). 

People have debated whether study or work at an active workstation influences cognitive ability (e.g., executive functions) and further interferes with working performance and efficiency [22]. Executive functions are a set of higher-order cognitive processes that are needed by people to perform the activities of daily living. They mainly consist of inhibitory control, working memory, cognitive flexibility, planning, and reasoning [23]. Furthermore, executive functions play a critical role in academic and professional success [23], socio-emotional development [24], and physical and mental health [25]. Some studies demonstrated that working at an active workstation did not adversely affect executive function performance [26,27,28,29,30], whereas others demonstrated a positive effect using active workstations [31], or they observed a selective impairment on a subdomain of executive functions [32]. It is worth noting that previous studies mainly focused on the end-state effects of using active workstations on behavioral performance of executive functions, rather than the in-task neural cognitive processes [28]. Functional near-infrared spectroscopy (fNIRS), a non-invasive technique to monitor the changes of concentration of oxygenated (oxy-Hb) and deoxygenated (deoxy-Hb) hemoglobin in the blood, is commonly used to assess cerebral activity. Recent evidence suggests that fNIRS is suitable for measuring the real-time changes in brain activity during exercise [33]. 

Therefore, to clarify the effects of using active workstations on executive function performance and brain activity, the fNIRS approach was used to measure the cortical activity during executive function tasks, performed by Chinese university students while cycling at an active workstation. 

## 2. Materials and Methods

### 2.1. Participants

Forty-one right-handed participants were recruited from two universities in Shanghai, China. All participants were undergraduate students or master’s students. Among the participants, one was excluded due to abnormal color vision and three participants did not complete the study protocol. Finally, 37 participants who completed the full protocol were included for data analysis. Written informed consents were obtained from all participants. The study protocol was approved by the ethical committee at Shanghai Jiao Tong University (ethical code: 20170100). The study was conducted in accordance with the ethical requirement of the Declaration of Helsinki. 

### 2.2. Procedures

**Figure 1:**
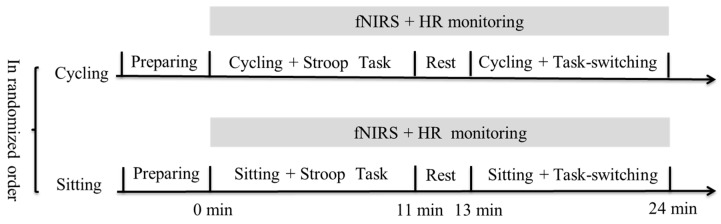
Chart of experimental design and protocol.

The study adopted a randomized crossover design. The experimental design and protocol are shown in Figure 1. Participants reported to the laboratory on two separate occasions, and each occasion was separated by at least 48 hours. At one experimental session, the participants performed the sitting condition, in which they completed two executive function tasks (i.e., Stroop color and word test, and task-switching). In this condition, the participants sat at an active cycling workstation (Loctek V9, Loctek Ergonomic Technology Corp., Ningbo, China) without performing any pedaling. At the other experimental session, the participants performed the cycling condition, and they completed the same cognitive task while cycling. A specific workload was not assigned, and therefore participants were allowed to cycle at a speed that was comfortable to them. The Borg rating of perceived exertion (RPE) scale was used to measure perceived exertion before and after the experimental sessions. The heart rate (HR) of participants was monitored throughout the two experimental sessions using a HR monitor (Suunto M5, SUUNTO Inc., Vantaa, Finland). In order to avoid potential order effects, participants performed the two experimental session in a counterbalanced order.

### 2.3. Measurements of Executive Functions

The participants’ performance on executive functions were measured by a computerized Stroop color and word test, and a task-switching paradigm. The two tasks were programmed using E-Prime 2.0 (Psychology Software Tools, Inc., Sharpsburg, PA, USA) in an event-related design. 

#### 2.3.1. Stroop Color and Word Test

The computerized Stroop task was programmed according to previous studies based on fNIRS [34]. For each trial, two rows of stimuli were displayed at the center of the screen. The bottom row displayed a Chinese word (i.e., red, green, blue, or yellow) printed in white, and the upper row displayed a Chinese word (neutral word or color word) printed in four colors (red, green, blue, or yellow). Two experimental conditions (neutral and incongruent) were defined in the present study based on the feature of upper stimuli. For the neutral condition, the upper row was a neutral word and the meaning of the word was not related to the color. For the incongruent condition, the upper row was a color word, and the color of the word did not denote the meaning of the word (e.g., the word “yellow” printed in red). Participants were instructed to decide whether the color of the upper row corresponded to the meaning of the bottom row. The Stroop test consisted of 48 judgment trials (24 neutral trials and 24 incongruent trials). For each trial, the fixation presentation varied randomly between 400 and 600 ms. Each stimulus was displayed on the screen for 400 ms, and then the response screen was displayed for 1600 ms, with an interval blank screen displayed for 11,000 ms. The order of the trials was randomly presented. 

In addition to the accuracy and the reaction time (RT), the Stroop interference effects were derived by the contrast of RT between the incongruent condition and the neutral condition [35]. 

#### 2.3.2. Task-Switching

The computerized task-switching paradigm adopted in this study was based on a previous study [36]. On each trial, either a red or green number (1–9, exclude 5) was displayed at the center of the screen. For the red number trials, participants were asked to press “X” on the keyboard if the number was smaller than 5 and to press the key “M” if the number was larger than 5. For the green number trials, participants were asked to press the key “X” if an odd number was presented and to press the key “M” if an even number was presented. The formal test began with the pure task (20 trials) in which the “large or small” and “odd or even” trials were respectively displayed. The participants were to maintain their response pattern in a task sequence. Following the pure task, the mixed task (32 trials) was displayed where the “large or small” and “odd or even” trials alternated after every second trial (in ABBA sequence). Therefore, the participants switched their response pattern in 16 trials, and they maintained their response pattern in the other trials. The changes in response pattern created a behavioral or cognitive cost between the non-switch condition and the switch condition, which is referred to as the global (mixed task vs. pure task) or local (switch trials in mixed task vs. non-switch trial in the mixed task) switch costs [37,38]. For each trial, the fixation presented varied randomly between 400 and 600 ms. The stimulus was displayed on the screen for 200 ms, and then the response screen was displayed for 1800 ms, with an interval blank screen was displayed for 10,000 ms. The stimuli’s digit was randomly presented. 

### 2.4. fNIRS Measurements and Data Preprocessing

**Figure 2:**
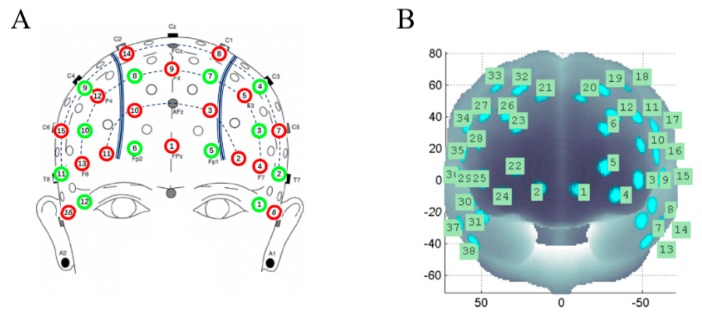
Location of the functional near-infrared spectroscopy (fNIRS) probe. (**A**) Spatial profiles of optical sources (red circles) and detector probes (green circles); (**B**) Estimated position of each channel.

The multi-channel continuous wave fNIRS system NIRScout (NIRX Medical Technologies LLC, New York, NY, USA, Sampling rate = 3.91 Hz) was used to monitor hemodynamic responses. Sixteen dual-wavelength source probes (760 and 850 nm) and 12 optical detector probes covered several 10–10 electroencephalography (EEG) positions. A nearby source-detector was paired to obtain 38 channels, and the distance between them was 3 cm. On the basis of the probabilistic estimation method [39,40], the projection of each channel in the brain region was estimated (Figure 2). The MNI data of all coordinates and the image reconstruction software package was obtained from Jichi medical university (http://www.jichi.ac.jp/brainlab/tools.html). 

The Matlab-based open-source software package HOMER2 v. 2.3 (https://homer-fnirs.org/) was used to process the fNIRS data [41]. The modified Beer–Lambert Law was used to convert optical density data into hemoglobin signals in an arbitrary unit. Raw data were bandpass filtered between 0.04 Hz and 0.3 Hz to remove baseline drift and physiological noise (e.g., HR). Since oxy-Hb signals have a better signal-to-noise ratio than deoxy-Hb, and deoxy-Hb signals have not shown significant Stroop interference effects in some fNIRS studies [42,43], only the oxy-Hb signals were used to monitor regional cortex activities [44]. The mean values of the oxy-Hb signals of the baseline (0–2 s before the onset of the trial) and the task (Stroop task: 4–13 s after the onset of the trial; task-switching: 4–12 s after the onset of the trial) were computed for each subject, channel, and task condition. In addition, individual hemodynamic responses were adjusted to the mean value of the baseline.

### 2.5. Anthropometrics and Physical Activity

The participants’ body weight and height were measured using standard protocols. Body composition was measured using an Inbody 720 Body Composition Analyzer (InBody Bldg., Seoul, Korea). The participants’ daily physical activity level was surveyed using the short form of an International Physical Activity Questionnaire (IPAQ-SF) [45]. The metabolic equivalent (MET) scores were calculated by standardized procedures. 

### 2.6. Statistics

The statistical analyses were performed using IBM SPSS software for Windows. Assumptions of data normality were examined graphically. The potential differences in the participants’ characteristics were tested by an unpaired sample t-test. The performances on the behavioral outcomes (i.e., accuracy and RT) and the fNIRS data were first assessed by a repeated-measures analysis of variance (RM-ANOVA). The paired sample t-test was conducted to assess the potential interaction effects between experiment and task conditions. For the Stroop interference effects and the switch costs (global and local), a paired sample t-Test was conducted between the experimental conditions (sitting/cycling). A false discovery rate (FDR) based on multiple testing adjustments was applied to the fNIRS data [46]. The statistical significance levels for all comparisons were set at *P* < 0.05 (two-sided).

## 3. Results

### 3.1. Participants Characteristics and Physiological Parameters

The participants’ characteristics were summarized by gender, shown in Table 1. The males were taller than the females, and the females had greater body fat percentage (all *P* < 0.05). 

The cycling condition resulted in an increased HR (Stroop task: 81.1 ± 1.8 BPM vs. 73.3 ± 1.8 BPM, *P* = 0.002; task-switching: 83.8 ± 1.8 BPM vs. 75.1 ± 1.3 BPM, *P* < 0.001) compared with the sitting condition. However, the RPE scores were not significantly different between the cycling and the sitting condition at pre-test (10.0 ± 2.7 vs. 10.0 ± 2.8, *P* = 0.80) and post-test (12.5 ± 2.5 vs. 12.5 vs. 2.2, *P* = 0.76). 

### 3.2. Behavioral Results

#### 3.2.1. Stroop Task

**Figure 3:**
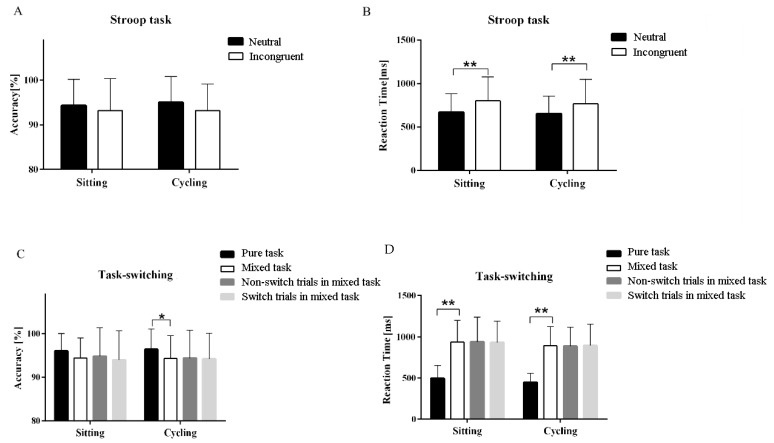
Behavioral results of the Stroop task and the task-switching (**A**) accuracy of Stroop task, (**B**) reaction time of Stroop task, (**C**) accuracy of task-switching, and (**D**) reaction time of task-switching. Data are expressed as mean ± SD. * indicates *P* < 0.05; ** indicates *P* < 0.01).

A RM-ANOVA performed on the accuracy of the Stroop task revealed that there were no significant main interaction effects. For RT, the RM-ANOVA revealed the main effects of the task condition (F (3, 108) = 37.25, *P* < 0.05), indicating that the RT of the neutral task was significantly smaller than that of the incongruent task (Figure 3). These results indicated that the Stroop interference effects were observed between the neutral task and the incongruent task. Post hoc analyses revealed that, in the sitting condition, the neutral task did not have greater accuracy than the incongruent task (94.31 ± 5.84% vs. 93.19 ± 7.13%, *P* = 0.32). However, the RT of the neutral task was significantly smaller than that of the incongruent task (670.55 ± 214.80 ms vs. 800.48 ± 274.80 ms, *P* < 0.001). In the cycling condition, the accuracy was not significantly different between the neutral task and the incongruent task (95.00 ± 5.83% vs. 93.19 ± 8.94%, *P* = 0.07). The RT of the neutral task was significantly smaller than that of the incongruent task (652.05 ± 203.52 ms vs. 766.76 ± 280.40 ms, *P* < 0.001).

The Stroop interference effects were derived and used to examine the potential differences between the experimental conditions. There were no significant differences in the Stroop interference effects between the sitting condition and the cycling condition (114.70 ± 141.99 ms vs. 129.93 ± 124.10 ms, *P* = 0.66).

#### 3.2.2. Task-Switching

As shown in Figure 3, the RM-ANOVA performed on the accuracy of task-switching revealed that there were no significant main interaction effects. For RT, the RM-ANOVA demonstrated significant main effects of the task condition (F (3, 108) = 172.82, *P* < 0.05), indicating that the RT of the pure task was significantly smaller than that of mixed task. Post-hoc analyses demonstrated that, in the sitting condition, the accuracy of the pure task was not significantly higher than that of the mixed task. The RT of the pure task was significantly smaller than that of the mixed task (497.27 ± 154.09 ms vs. 936.24 ± 264.12 ms, *P* < 0.001). In the cycling condition, the accuracy of the pure task was significantly higher than that of the mixed task (96.36 ± 4.67% vs. 94.27 ± 5.26%, *P* = 0.03). The RT of the pure task was significantly smaller than that the mixed task (446.25 ± 111.56 ms vs. 891.22 ± 232.56 ms, *P* < 0.001).

There were no significant differences in the local switch costs (−8.20 ± 175.71 ms vs. 9.73 ± 155.90 ms, *P* = 0.67) and the global switch costs (438.98 ± 203.61 ms vs. 444.97 ± 174.43 ms, *P* = 0.90) between the sitting and the cycling conditions. 

### 3.3. fNIRS Results

#### 3.3.1. Stroop Task

The RM-ANOVA performed on channel-wise oxy-Hb concentrations revealed significant main effects of the experimental condition in channel 5 (F (1, 36) = 5.24, *P* < 0.05, FDR-corrected). The RM-ANOVA also revealed significant main interaction effects of the experimental conditions (sitting/cycling) × task condition in channel 5 (F (1, 36) = 4.60, *P* < 0.05, FDR-corrected); channel 10 (F (1, 36) = 5.50, *P* < 0.05, FDR-corrected); and channel 12 (F (1, 36) = 5.29, *P* < 0.05, FDR-corrected). Post hoc analyses demonstrated that the oxy-Hb concentrations in response to the incongruent task in channel 10 were significantly higher than the neutral task (channel 10: 0.76 ± 0.93 vs. 1.22 ± 1.29, *P* = 0.02, FDR-corrected) in the sitting condition. In the cycling condition, there were no significant differences in oxy-Hb responses between the neutral task and the incongruent task. 

**Figure 4:**
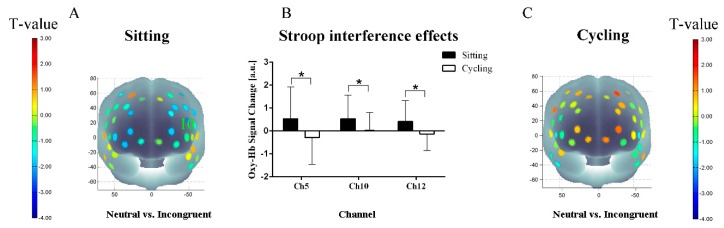
fNIRS results of the Stroop task (**A**) t-map of oxygenated hemoglobin (oxy-Hb) concentrations in the sitting condition; (**B**) Oxy-Hb changes related to the Stroop interference effects; (**C**) t-map of oxy-Hb concentrations in the cycling condition. Data are expressed as mean ± SD. * indicates FDR-corrected *P* < 0.05).

In channel 5, 10, and 12 (Figure 4), the cycling condition resulted in lower oxy-Hb associated with the Stroop interference effects (oxy-Hb interference) than that of the sitting condition channel 5 (0.52 ± 1.42 vs. −0.29 ± 1.16, *P* < 0.05, FDR-corrected); channel 10 (0.54 ± 1.03 vs. 0.01 ± 0.80, *P* < 0.05, FDR-corrected); and channel 12 (0.42 ± 0.91 vs. −0.13 ± 0.72, *P* < 0.05, FDR-corrected). The three channels were located approximately at the frontopolar area and the left-dorsolateral prefrontal cortex (L-DLPFC).

#### 3.3.2. Task-Switching

The RM-ANOVA performed on channel-wise oxy-Hb concentrations revealed significant main effects of the task condition in the following: channel 3 (F (3, 108) = 5.67, *P* < 0.05, FDR-corrected); channel 4 (F (3, 108) = 3.26, *P* < 0.05, FDR-corrected); channel 10 (F (3, 108) = 6.66, *P* < 0.05, FDR-corrected); channel 15 (F (3, 108) = 4.60, *P* < 0.05, FDR-corrected); channel 16 (F (3, 108) = 4.74, *P* < 0.05, FDR-corrected); channel 17 (F (3, 108) = 5.80, *P* < 0.05, FDR-corrected); channel 24 (F (3, 108) = 4.76, *P* < 0.05, FDR-corrected); and channel 31 ( F(3, 108) = 5.68, *P* < 0.05, FDR-corrected). A significant experiment condition (sitting/cycling) × task condition (pure/mixed) interaction was also observed in channel 3 (F (3, 108) = 2.89, *P* < 0.05, FDR-corrected); channel 18 (F (3, 108) = 4.94, *P* < 0.01, FDR-corrected); channel 29 (F (3, 108) = 6.14, *P* < 0.05, FDR-corrected); and channel 31(F (3, 108) = 5.95, *P* < 0.05, FDR-corrected). 

**Figure 5:**
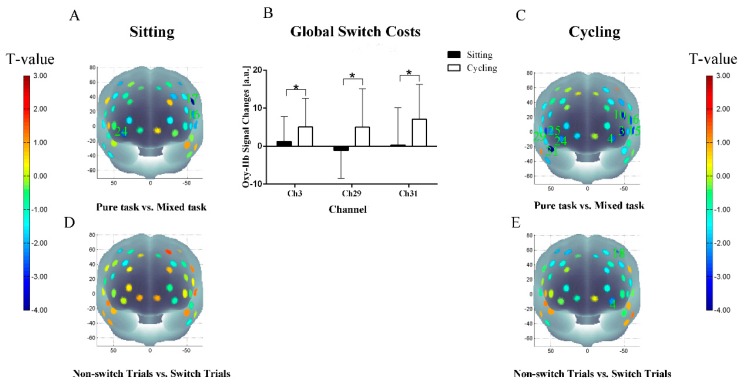
fNIRS results of task-switching (**A**) t-map of oxy-Hb concentrations in the sitting condition, pure task vs. mixed task; (**B**) Oxy-Hb changes related to the global switch costs; (**C**) t-map of oxy-Hb concentrations in the cycling condition, pure task vs. mixed task; (**D**) t-map of oxy-Hb concentrations in the sitting condition, non-switch trials vs. switch trials; (**E**) t-map of oxy-Hb concentrations in the cycling condition, non-switch trials vs. switch trials. Data are expressed as mean ± SD. * indicates FDR-corrected *P* < 0.05.

As shown in Figure 5, post hoc analyses demonstrated that the oxy-Hb concentrations in response to the mixed task and the pure task significantly differed in channels 16, 17, and 24 in the sitting condition (all *P* < 0.05, FDR-corrected). In the cycling condition, the oxy-Hb concentrations in response to the mixed task were significantly higher than the pure task in channels 3, 4, 10, 15, 16, 24, 25, 29, and 31 (all *P* < 0.05, FDR-corrected). Meanwhile, in the cycling condition, the oxy-Hb concentrations in response to the switch trials in channels 4 and 18, were higher than those in response to the non-switch trials (all *P* < 0.05, FDR-corrected).

As shown in Figure 5B, in channel 3 (approximately located in frontopolar area), channel 29 (approximately located in right ventrolateral prefrontal cortex, VLPFC), and channel 31 (approximately located in temporopolar area), the cycling condition resulted in a higher oxy-Hb concentration (change) associated with the global switch costs than with the sitting condition in channel 3 (1.26 ± 6.55 vs. 5.05 ± 7.48, *P* < 0.05, FDR-corrected); channel 29 (−1.11 ± 7.40 vs. 5.05 ± 10.11, *P* < 0.05, FDR-corrected); and channel 31 (0.33 ± 9.79 vs. 7.11 ± 9.14, *P* < 0.05, FDR-corrected). There were no significant differences in the local switch costs related to the oxy-Hb concentrations between the sitting and the cycling conditions.

## 4. Discussion

The present study aimed to determine whether self-paced cycling at an active workstation influences concurrent performance on executive functions and cortical activity in young adults. The study results demonstrated that the behavioral performances on the Stroop task and the task-switching paradigm were not influenced by cycling at an active workstation. The cortical activity pattern was changed when conducting two cognitive tasks while cycling at an active workstation. 

In the current study, the RT, the accuracy of the Stroop task, and the Stroop interference effects did not differ between the two experimental conditions. For the task-switching, the RT and the accuracy of the pure task and the mixed task, as well as the global switch costs and the local switch costs were not different between either of the two experimental conditions. The results indicated that self-paced exercise at an active workstation did not impair the concurrent performances on executive functions. It was hypothesized that there is an inverted-U relationship between exercise-induced arousal and cognitive performance [47]. In this study, self-paced cycling was performed at a low-intensity without an obvious increase in HR and RPE, which indicates that self-paced cycling did not lead to a significant increase in arousal. Therefore, the unchanged arousal levels may partly explain the reasons for the behavioral results. Although the cognitive measures are not the same, the behavioral results of the present study are in line with several previous studies. A study of young adults showed that slow walking (1.5 mph) on a treadmill desk did not affect concurrent performance on executive functions measured by the Eriksen flanker task and the go/no-go task [29]. Similarly, Ehmann et al. [28] found that, in young adults, self-selected low-intensity walking on an active workstation did not affect performance on the tests of inhibitory control, working memory, cognitive flexibility, and global executive functions. Pilcher et al. [30] reported that, in young college students, there were no differences on meta-cognitive measures between using an activity workstation with low-intensity cycling and using a traditional desk. It is also worth noting that some studies observed that the cognitive performance was influenced when using an active workstation. Interestingly, Torbeyns et al. [31] showed that, compared with a sitting condition, cycling at 30% maximal external power improved the performance of adults on the Stroop task. In contrast, Zhang et al. [32] investigated the effects of standing and walking on self-paced speed (2.3 km/h) and faster speed (3.5 km/h) on inhibitory control, working memory, and cognitive flexibility in young adults. Their study showed that walking on a workstation with self-paced speed or faster speed selectively impaired performance of working memory measured by the N-back task, with no influence on inhibitory control and cognitive flexibility. 

Although the behavioral performances did not differ between the sitting and the cycling conditions in the current study, it is important to clarify whether the in-task neural processing was affected when cycling using an active workstation. The fNIRS results showed that oxy-Hb concentrations associated with the Stroop interference effects and the global switch costs were different in some brain areas between the sitting and the cycling conditions. The results indicated that the dual-task condition (cycling condition) led to changes in cortical neural activity in relation to the Stroop task and the task-switching. Therefore, the findings suggest that the cognitive resources were re-allocated during the cycling condition. It is interesting that the pattern of changes in cortical activity differed between the Stroop task and the task-switching paradigm. The oxy-Hb concentrations associated with the Stroop interference in the three channels were lower during the cycling condition, which indicated a competition of cognitive resources when performing the Stroop task while cycling at an active workstation. The findings may be explained by the limited-capacity information processing model [48,49], which suggest that the attentional resource was re-allocated when performing two tasks simultaneously (in this study, a cognitive task and a motor task of cycling). In contrast to the Stroop task, the oxy-Hb concentrations associated with the global switch costs in three channels were higher during the cycling condition. This may be due to the fact that the event-related task-switching paradigm is not difficult for the young participants. People did not need much cognitive resource when performing the single cognitive task. However, performing task-switching while cycling leads to additional cognitive resource. Therefore, an increased cortical activity was observed during the cycling condition. It is not possible to directly compare our fNIRS results with others, since, to the best of our knowledge, no other studies examined the pattern of executive function-related cortical activation using fNIRS under the condition of cycling at an active workstation. However, a study [50], using EEG, investigated the effects of using an active treadmill desk (self-determined low-intensity speeds) on brain activation during an attention task. No significant differences of steady state visually evoked potentials (SSVEPs) were observed among the sitting, standing, and walking conditions. Obviously, more studies employing similar study designs are warranted to confirm the effects of using active workstations on brain activity and allocation patterns of cognitive resource. 

Given the fact that there is an increasing prevalence of sedentary lifestyle, the findings of this study have practical implications. Specifically, the implementation of active workstations in working places may not sacrifice or interfere with executive functioning, whereas it increases daily energy expenditure and breaks prolonged sedentary time. However, considering the fNIRS results of changed cortical activity patterns, this study also indicated that the cognitive resource was reallocated when performing cognitive tasks while self-paced cycling. Therefore, future studies employing various exercise intensities are warranted to elucidate the upper limit of exercise intensity at which the cognitive performance is not influenced by using an active workstation. Furthermore, future studies should investigate the influences of using active workstation on cognitive performances and neural activity patterns in various age groups. 

The primary strength of the study is that the pattern of cortical activation while performing cognitive tasks was measured. It allowed the present study to not only examine the effects of using an active workstation on cognitive task performances but also the in-task neural processing. However, the findings of the study should be interpreted within the context of some limitations. First, the study was conducted under a simulated working environment rather than actual office workplace. Therefore, the generalization of the findings on executive function performance to actual work efficiency may be limited. However, it is well documented that executive functions are important for success in many aspects of life, including academic performance and productivity at work [23]. Secondly, the study only examined the acute effects of using an active workstation for a short period of time. Therefore, future studies are needed to clarify the long-term effects of the implementation of active workstations.

## 5. Conclusions

The study indicated that the behavioral performances on executive functions were not affected by cycling at an active workstation. However, the in-task cortical neural activity was changed in some brain areas, which indicated that cognitive resources were reallocated during cycling at an active workstation.

## Figures and Tables

**Table 1:** Participants’ characteristics.

Characteristics	Men (*n* = 20)	Women (*n* = 17)	All (*n* = 37)	*P* for gender
**Age (years)**	21.0 ± 2.9	22.4 ± 1.6	21.6 ± 2.5	0.08
**Height (cm)**	174.4 ± 6.0	166.4 ± 5.3	170.7 ± 6.9	<0.001
**Weight (kg)**	64.0 ± 14.8	58.5 ± 8.1	61.4 ± 12.3	0.18
**BMI (kg/m2)**	22.1 ± 2.1	21.5 ± 3.1	21.8 ± 2.6	0.46
**Rest heart rate (BPM)**	76.2 ± 11.3	75.8 ± 10.3	76.0 ± 10.7	0.93
**Body fat percentage (%)**	17.6 ± 5.4	23.1 ± 5.8	20.2 ± 6.2	0.003
**Physical activity (MET-min/week)**	3021 (400–15,600)	2506 (480–5432)	2785 (400–15,600)	0.57

Note: BMI, body mass index; BPM, beats per minute; MET, metabolic equivalent. Data are expressed as mean ± standard deviation (SD) or median (interquartile range).
